# Function, Oxidative, and Inflammatory Stress Parameters in Immune Cells as Predictive Markers of Lifespan throughout Aging

**DOI:** 10.1155/2019/4574276

**Published:** 2019-06-02

**Authors:** Irene Martínez de Toda, Carmen Vida, Luis Sanz San Miguel, Mónica De la Fuente

**Affiliations:** ^1^Department of Genetics, Physiology and Microbiology (Unit of Animal Physiology), Faculty of Biology, Complutense University, Madrid, Spain; ^2^Institute of Investigation Hospital 12 Octubre, Madrid, Spain; ^3^Department of Statistics and Operational Research, Faculty of Mathematics, Complutense University, Madrid, Spain

## Abstract

According to the oxidative-inflammatory theory of aging, there is a link between the function, the oxidative-inflammatory stress state of immune cells, and longevity. However, it is unknown which immune cell parameters can predict lifespan and if there would be any changes in this prediction, depending on the age of the subject. Therefore, a longitudinal study in mice was performed analysing immune function (chemotaxis of macrophages and lymphocytes, phagocytosis of macrophages, natural killer (NK) activity, and lymphoproliferation capacity), antioxidant (catalase (CAT), glutathione peroxidase (GPx), and glutathione reductase (GR) activities as well as reduced glutathione (GSH) concentrations), oxidant (oxidized glutathione (GSSG), superoxide anion, and malondialdehyde (MDA) concentrations), and inflammation-related markers (basal release of IL-1*β*, IL-6, TNF-*α*, and IL-10) in peritoneal leukocytes from mice at the adult, mature, old, very old, and long-lived ages (40, 56, 72, 96, and 120 ± 4 weeks of age, respectively). The results reveal that some of the investigated parameters are determinants of longevity at the adult age (lymphoproliferative capacity, lymphocyte chemotaxis, macrophage chemotaxis and phagocytosis, GPx activity, and GSH, MDA, IL-6, TNF-*α*, and IL-10 concentrations), and therefore, they could be proposed as markers of the rate of aging. However, other parameters are predictive of extreme longevity only at the very old age (NK activity, CAT and GR activities, and IL-6 and IL-1*β* concentrations), and as such, they could reflect some of the adaptive mechanisms underlying the achievement of high longevity. Nevertheless, although preliminary, the results of the present study provide a new perspective on the use of function, redox, and inflammatory parameters in immune cells as prognostic tools in aging research and represent a novel benchmark for future work aimed at prediction of lifespan.

## 1. Introduction

Nowadays, increasing average life expectancy is turning the focus of gerontologists from trying to increase lifespan to experiencing healthy aging by prolonging the so-called “healthspan” [1]. It has been predicted that in 2050, 22% of the world population will be over 60 years old. Thus, many countries are facing an increased prevalence of age-related diseases and increasing healthcare costs given that the rapid rise in older people is accompanied by an increase in the number of subject with chronic age-related diseases, such as heart disease, lung disease, stroke, cancer, and diabetes [2]. However, some individuals live more than a century without ever suffering from the chronic diseases that afflict most humans much earlier in their lives. Therefore, focus should be placed on the study of those successful phenotypes to assist in the understanding of aging. Thus, centenarians have been shown to retain independence and capability as well as cognition at higher levels for longer than the general population, together with postponed mortality [2–5]. Therefore, their study might help to achieve an extended healthy lifespan for the wider population.

Several theories have been proposed to explain the aging process. The oxidative-inflammatory theory of aging [6] links the age-related increase in oxidative stress [7, 8] with the chronic low-grade inflammation, the so-called “inflamm-aging” [9], through the interplay of the immune system. It is known that the age-related increase in oxidative stress impairs the correct functioning of cells. Given that oxidation and inflammation are interlinked processes, the increase in oxidative stress in immune cells results in an increased release of proinflammatory mediators, giving as a result the age-related establishment of a chronic oxidative and inflammatory stress [6]. According to this theory, a relationship has been found between the oxidative and inflammatory states of immune cells, their functional capacity, and the lifespan of a subject [6]. In this regard, it has been demonstrated that centenarians have immune cell function and redox parameters similar to those in adults, despite their advanced age [10, 11]. However, if they maintain this optimal functionality during their whole lifespan or they undergo some remodelling of these parameters during aging is unknown. Therefore, a deep understanding of these subjects would require their follow-up throughout the aging process to shed light into which changes or adaptations are the “successful ones.” Since a longitudinal study is impossible to carry out in human subjects throughout the whole aging process, mice, which have a mean longevity of 2 years, were used for this work. In addition, peritoneal leukocytes were chosen as a sample of study given that they can be extracted without killing the mouse and in the absence of anaesthesia. This fact allowed the monitoring of mice from the adult age until the natural death of the animals.

Thus, a longitudinal study was performed analysing several functions (macrophage chemotaxis and phagocytosis, natural killer activity, lymphocyte chemotaxis, and lymphoproliferation capacity), redox parameters (catalase, glutathione peroxidase, and glutathione reductase activities, reduced and oxidized glutathione, and superoxide anion and malondialdehyde concentrations), and inflammatory mediators (basal release of IL-6, IL-1*β*, TNF-*α*, and IL-10) in peritoneal leukocytes throughout the aging process of mice. This approach allowed us to address three important questions: (i) which markers are the most important predictors of remaining longevity in adult or middle life? (ii) Are the same parameters predictive of successful aging at very advanced age? (iii) Which changes or adaptations an individual experiences throughout his/her lifetime that allow the attainment of extreme longevity?

## 2. Material and Methods

### 2.1. Experimental Animals and Extraction of Peritoneal Leukocytes

40 female outbred ICR/CD1 exreproductive mice (*Mus musculus*) were acquired from Janvier Labs (Germany) when they were 32 ± 4 weeks old. The collection of peritoneal suspensions was performed at the adult (40 ± 4 weeks; *n* = 38), mature (56 ± 4 weeks; *n* = 25), old (72 ± 4 weeks; *n* = 15), very old (96 ± 4 weeks; *n* = 11), and long-lived (120 ± 4 weeks; *n* = 3) ages. Mice were further divided at each age point into adult mice (survivors, *n* = 22; nonsurvivors, *n* = 13; and long-lived, *n* = 3); mature mice (survivors, *n* = 12; nonsurvivors, *n* = 10; and long-lived, *n* = 3); old mice (survivors, *n* = 8; nonsurvivors, *n* = 4; and long-lived, *n* = 3); and very old mice (survivors, *n* = 3; nonsurvivors, *n* = 8). All the animals had a natural death, whereas when no weight loss (<20%), moribund state, or tumor formation was detected, the cause of death was not further investigated. Leukocytes from peritoneal suspensions were identified by their morphology (macrophages or lymphocytes) and quantified (number of cells/mL) in Neubauer chambers. The measurement of markers was performed using unfractionated peritoneal leukocytes to better reproduce the *in vivo* situation. The peritoneal suspensions were adjusted to a specific number of macrophages, lymphocytes, or total leukocytes, depending on the parameter analysed as described in the corresponding section.

### 2.2. Analysis of Immune Function Parameters

#### 2.2.1. Chemotaxis

Cell suspensions were adjusted to 0.5 × 10^6^ cells (macrophages or lymphocytes)/mL in Hank's medium and placed into a Boyden chamber. The number of cells that migrated towards formyl-Met-Leu-Phe was counted and expressed as the Chemotaxis Index, as previously described [11].

#### 2.2.2. Phagocytosis

Cell suspensions were adjusted to 0.5 × 10^6^ macrophages/mL in Hank's medium and placed into migration inhibition factor (MIF) plates for 30 min. After washing, latex beads were added into the plates and the number of latex beads ingested by 100 macrophages was counted and expressed as the Phagocytic Index, as previously described [11].

#### 2.2.3. Natural Killer (NK) Cytotoxicity

Cell suspensions were adjusted to 10^6^ total cells/mL in RPMI 1640 medium and placed into 96-well plates. Murine YAC-1 lymphoma cells were added into wells, and NK activity was assessed by quantifying released lactate dehydrogenase into the medium (Cytotox 96 TM, Promega, Germany). The results were expressed as the percentage of tumor cells killed (% lysis), as previously described [11].

#### 2.2.4. Lymphoproliferative Capacity

Cell suspensions were adjusted to 0.5 × 10^6^ lymphocytes/mL in RPMI 1640 medium supplemented with fetal bovine serum (FBS) and placed into 96-well plates. The mitogen concanavalin A (Con A) or complete medium was added into wells and incubated for 48 h. Then, ^3^H-thymidine was also added and incubated for 24 h. ^3^H-Thymidine uptake was quantified in a beta counter both in basal and stimulated conditions, and results were expressed as lymphoproliferation capacity (%), 100% being the counts per minute (cpm) in basal conditions, as previously described [11].

### 2.3. Determination of Redox Parameters

#### 2.3.1. Catalase (Cat) Activity

Cell suspensions were adjusted to 10^6^ total cells/mL in Hank's medium and centrifuged, and cell pellets were resuspended in oxygen-free phosphate buffer 50 mM. Then, they were sonicated and supernatants were used for the enzymatic reaction together with 14 mM H_2_O_2_ as substrate. Decomposition of H_2_O_2_ was measured at 240 nm as previously described [12]. The results were expressed as units (U) of catalase activity/mg protein.

#### 2.3.2. Glutathione Peroxidase (GPx) Activity

Cell suspensions were adjusted to 10^6^ total cells/mL in Hank's medium and centrifuged, and cell pellets were resuspended in oxygen-free phosphate buffer 50 mM. Then, they were sonicated and supernatants were used for the enzymatic reaction together with cumene hydroperoxide as a substrate (cumene-OOH) as previously described [12]. Oxidation of NADPH was measured at 340 nm. The results were expressed as units (U) of GPx activity/mg protein.

#### 2.3.3. Glutathione Reductase (GR) Activity

Cell suspensions were adjusted to 10^6^ total cells/mL in Hank's medium and centrifuged, and the cell pellets were resuspended in oxygen-free phosphate buffer 50 mM and EDTA 6.3 mM. Then, they were sonicated and supernatants were used for the enzymatic reaction together with oxidized glutathione (GSSG) 80 mM as substrate, as previously described [12]. Oxidation of NADPH was measured at 340 nm. The results were expressed as units (U) of GR activity/mg protein.

#### 2.3.4. Glutathione Concentration

Cell suspensions were adjusted to 10^6^ total cells/mL in Hank's medium and centrifuged, and cell pellets were resuspended in phosphate buffer 50 mM and EDTA 0.1 M, pH 8. Then, they were sonicated and supernatants were used for the quantification of both reduced (GSH) and oxidized (GSSG) glutathione by the reaction capacity that GSSG and GSH have with o-phthalaldehyde (OPT) at pH 12 and pH 8, respectively, resulting in the formation of a fluorescent compound, as previously described [12]. Fluorescence was measured at 350 nm excitation and 420 nm emission. Results were expressed as nmol of GSSG and GSH per milligram of protein. Moreover, the GSSG/GSH ratio was calculated for each sample.

#### 2.3.5. Intracellular Superoxide Anion Concentration

Cell suspensions were adjusted to 10^6^ macrophages/mL in Hank's medium and mixed with nitroblue tetrazolium (NBT) (1 mg/mL). After 60 min incubation, the reaction was stopped with HCl 0.5 M; samples were centrifuged and supernatants discarded. Intracellular reduced NBT was extracted with dioxan and absorbance was measured at 525 nm, as previously described [12]. Results were expressed as nmol superoxide anion/10^6^ macrophages.

#### 2.3.6. Malondialdehyde (MDA) Concentration

Determination of MDA concentration was evaluated using the commercial kit “Lipid Peroxidation (MDA) Assay Kit” (BioVision, CA, USA). Cell suspensions were adjusted to 10^6^ total cells/mL in Hank's medium and centrifuged, and cell pellets were resuspended in MDA lysis buffer. Then, they were sonicated and supernatants were incubated with thiobarbituric acid (TBA) for 60 min in a water bath. Then, samples were centrifuged and supernatants collected and dispensed into 96-well plates for spectrophotometric measurement at 532 nm. Results were expressed as nmol MDA/mg protein.

### 2.4. Determination of Inflammatory Parameters

#### 2.4.1. Cytokine Measurement

After incubation of peritoneal immune cells for 48 hours in the absence of any mitogen (basal conditions), supernatants were collected. The basal release of IL-1*β*, IL-6, TNF-*α*, and IL-10 was measured simultaneously in these supernatants by multiplex luminometry (Beadlyte mouse multiplex cytokine detection system, MHYSTOMAG-70K, Upstate, Millipore).

### 2.5. Statistical Analysis

Differences between groups of the same age were studied using Student's *t*-test for independent samples. Age-related differences in the group of long-lived mice were studied using Student's *t*-test for paired samples. Two-sided *P* < 0.05 was considered the minimum level of significance. In order to investigate the potential role of the parameters studied as predictors of lifespan, Pearson's correlation coefficients were calculated for each of the parameters studied at each age and the respective lifespan that each individual mouse achieved.

## 3. Results

Several immune function, redox, and inflammatory parameters were assessed in peritoneal leukocytes from female mice, throughout a longitudinal study, starting at the age of 40 weeks until the natural death of the animals. Each mouse was monitored individually along its aging process. At each age analysed, the results are shown classifying mice into three groups: those that lived until the next age point studied, those that died before the next age point, and the animals that reached extreme longevity.

**Figure 1:**
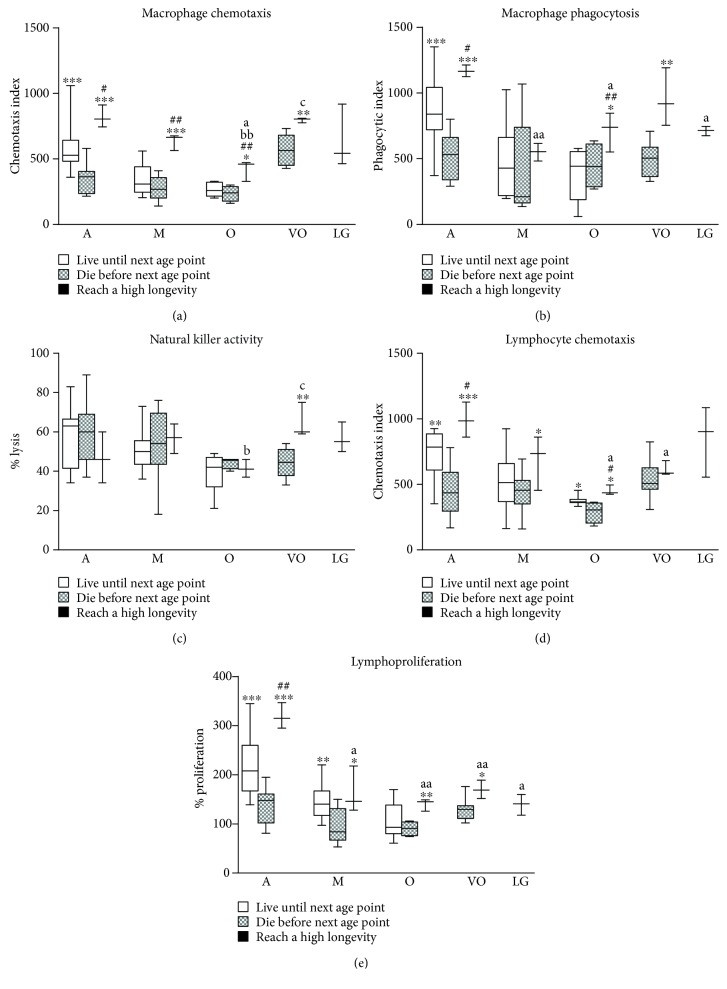
Immune function parameters analysed in peritoneal leukocytes from mice throughout a longitudinal study: (a) macrophage chemotaxis; (b) macrophage phagocytosis; (c) natural killer activity; (d) lymphocyte chemotaxis; (e) lymphoproliferation. A: adult mice (40 weeks old; *n* = 38); M: mature mice (56 weeks old; *n* = 25); O: old mice (72 weeks old; *n* = 18); VO: very old mice (96 weeks old; *n* = 11); LG: long-lived mice (120 weeks old; *n* = 3). ^∗^*P* < 0.05; ^∗∗^*P* < 0.01; ^∗∗∗^*P* < 0.001 with respect to the group of mice that die before the next age point (Student's *t*-test for independent samples). a: *P* < 0.05, aa: *P* < 0.01 with respect to adult mice that reach extreme longevity; b: *P* < 0.05, bb: *P* < 0.01 with respect to mature mice that reach extreme longevity; c: *P* < 0.05 with respect to old mice that reach extreme longevity (Student's *t*-test for paired samples).

Regarding immune function parameters (Figure 1), mice of the group that survived until the next age point show, in general, a better immune functionality than those that die. However, differences exist depending on the parameter and the age point investigated. Accordingly, mice that reach extreme longevity also show better immune functions, in general, compared to those that die and in some cases even better than those that live to the next age point studied. When focusing on the age-related changes that those mice that reach extreme longevity undergo, it was found that they experience a decrease in almost all immune functions when they are old followed by a subsequent increase at the very old age, showing optimal immune function values when they are long-lived.

**Figure 2:**
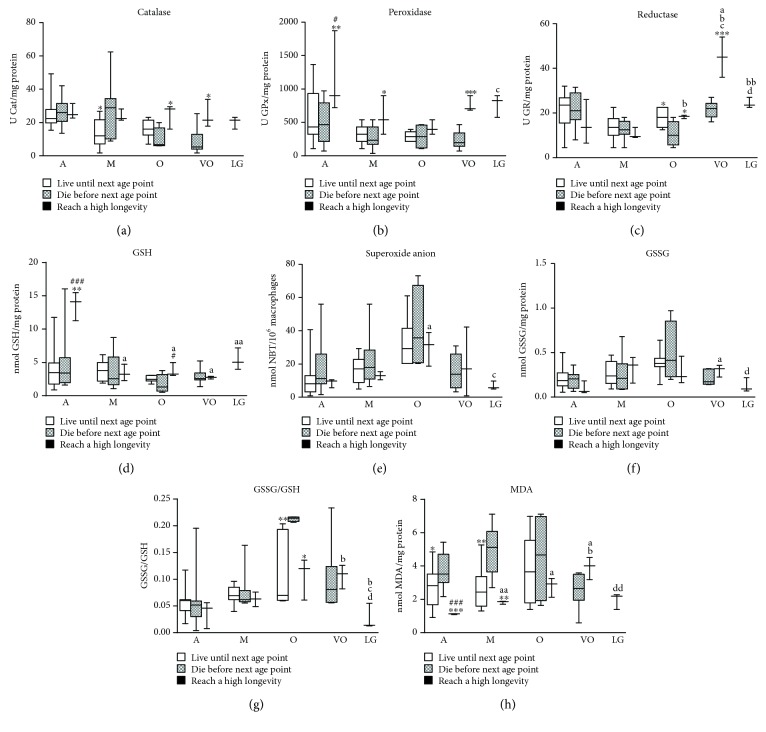
Redox parameters analysed in peritoneal leukocytes from mice throughout a longitudinal study: (a) catalase (Cat) activity; (b) glutathione peroxidase (GPx) activity; (c) glutathione reductase (GR) activities; (d) reduced glutathione (GSH) concentration; (e) superoxide anion concentration; (f) oxidized glutathione (GSSG) concentration; (g) GSSG/GSH ratio; (h) MDA concentration. A: adult mice (40 weeks old; *n* = 38); M: mature mice (56 weeks old; *n* = 25); O: old mice (72 weeks old; *n* = 18); VO: very old mice (96 weeks old; *n* = 11); LG: long-lived mice (120 weeks old; *n* = 3). ^∗^*P* < 0.05; ^∗∗^*P* < 0.01; ^∗∗∗^*P* < 0.001 with respect to the group of mice that die before the next age point (Student's *t*-test for independent samples). a: *P* < 0.05, aa: *P* < 0.01 with respect to adult mice that reach high longevity; b: *P* < 0.05, bb: *P* < 0.01 with respect to mature mice that reach high longevity; c: *P* < 0.05 with respect to old mice that reach high longevity; d: *P* < 0.05, dd: *P* < 0.01 with respect to very old mice that reach high longevity (Student's *t*-test for paired samples).

With respect to antioxidant parameters (Figures 2(a)–2(d)), there are almost no differences between those mice that survived until the next age point and those that died. However, the group of mice that reach extreme longevity shows a higher antioxidant capacity, in general, compared to those that die at most of the ages studied. This group of mice that reach high longevity experience an age-related decrease in GSH concentration compared to when they were adults, but they undergo a large increase in GPx and GR activities when they are close to reaching extreme longevity.

In relation to oxidant parameters (Figures 2(e)–2(h)), the group of mice that survive to the next age and those that become long-lived show lower GSSG/GSH ratios and MDA concentrations than those that die. Long-lived mice experience an increase in almost all oxidant parameters when they are old and very old, followed by a subsequent decrease, and thus showing optimal levels when they are long-lived.

**Figure 3:**
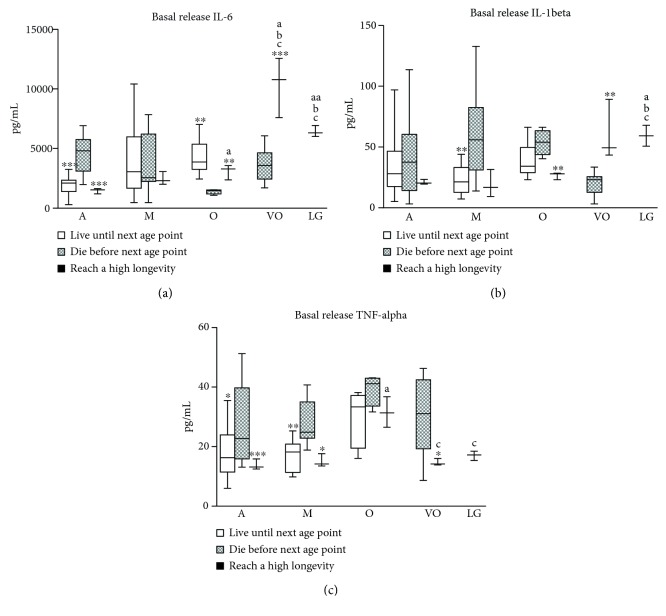
Basal release of cytokines in peritoneal leukocytes from mice throughout a longitudinal study: (a) IL-6 concentration (pg/mL); (b) IL-1*β* concentration (pg/mL); (c) TNF-*α* concentration (pg/mL); (d) IL-10 concentration (pg/mL). A: adult mice (40 weeks old; *n* = 38); M: mature mice (56 weeks old; *n* = 25); O: old mice (72 weeks old; *n* = 18); VO: very old mice (96 weeks old; *n* = 11); LG: long-lived mice (120 weeks old; *n* = 3). ^∗^*P* < 0.05; ^∗∗^*P* < 0.01; ^∗∗∗^*P* < 0.001 with respect to the group of mice that die before the next age point (Student's *t*-test for independent samples). a: *P* < 0.05, aa: *P* < 0.01 with respect to adult mice that reach high longevity; b: *P* < 0.05 with respect to mature mice that reach high longevity; c: *P* < 0.05 with respect to old mice that reach high longevity (Student's *t*-test for paired samples).

Regarding inflammatory mediators (Figure 3), both the groups of mice that survive to the next age and those that reach high longevity show, at most of the ages studied, lower basal release of proinflammatory cytokines and higher release of the anti-inflammatory cytokine IL-10, compared to those that die. Those mice that become long-lived experience a gradual increase in IL-6, IL-1*β*, and TNF-*α* when they age, but they lessen TNF-*α* basal release when they are long-lived. In the same way, they experience a slight decrease in IL-10 basal release when they age, followed by a large increase when they are long-lived.

Due to the observed heterogeneity of values for a given parameter among mice from the same age group and given that the lifespan of each mouse was monitored individually, it was possible to investigate the relationship between the values of a given parameter of each mouse at a certain age and its final achieved lifespan.

**Figure 4:**
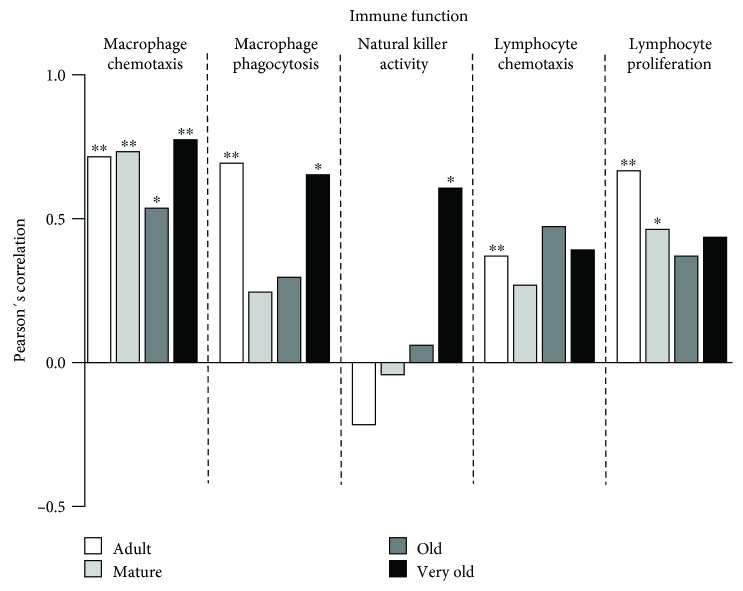
Pearson's correlation coefficient for each immune function parameter and lifespan at each age. Adult mice: 40 weeks old. Mature mice: 56 weeks old. Old mice: 72 weeks old. Very old mice: 96 weeks old. ^∗^*P* < 0.05; ^∗∗^*P* < 0.01; ^∗∗∗^*P* < 0.001.

With respect to immune function parameters (Figure 4), a positive correlation was found between the chemotaxis capacity of macrophages and lifespan at the adult, mature, old, and very old ages (*P* < 0.01; *P* < 0.01; *P* < 0.05; and *P* < 0.01, respectively) and between the phagocytic ability of macrophages and achieved lifespan at the adult and very old ages (*P* < 0.01; *P* < 0.05, respectively). In addition, positive correlations were also found between the NK activity and remaining lifespan at the very old age (*P* < 0.05), between the chemotaxis capacity of lymphocytes and lifespan at the adult age (*P* < 0.01), and between the ability of lymphocytes to proliferate in response to Con A and achieved lifespan at the adult and mature ages (*P* < 0.01; *P* < 0.05, respectively).

**Figure 5:**
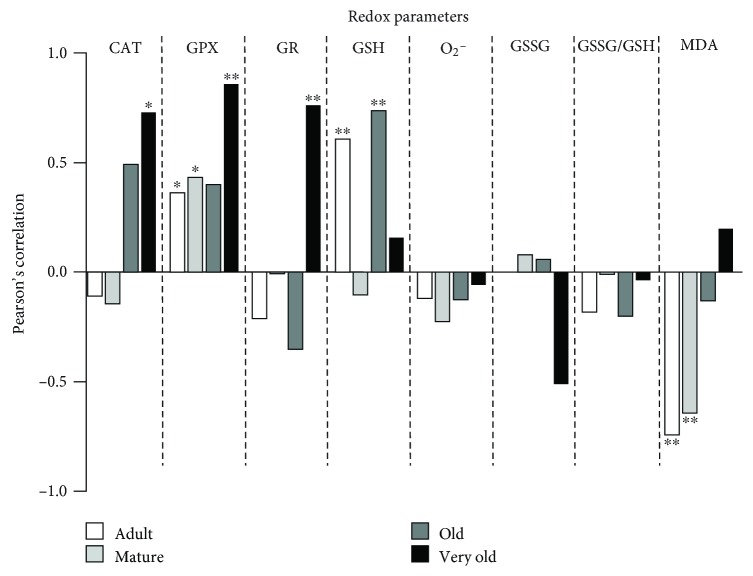
Pearson's correlation coefficient for each redox parameter and lifespan at each age. Adult mice: 40 weeks old. Mature mice: 56 weeks old. Old mice: 72 weeks old. Very old mice: 96 weeks old. ^∗^*P* < 0.05; ^∗∗^*P* < 0.01; ^∗∗∗^*P* < 0.001.

Regarding redox parameters (Figure 5), there are positive correlations between Cat and GR activities and lifespan at the very old age (*P* < 0.05; *P* < 0.01, respectively) and between GPx activity and lifespan at the adult, mature, and very old ages (*P* < 0.05; *P* < 0.05; and *P* < 0.01, respectively). In addition, a positive correlation was found between GSH concentration and lifespan at the adult and old ages (*P* < 0.01), whereas a negative correlation was found between MDA concentration and lifespan at the adult and mature ages (*P* < 0.01).

**Figure 6:**
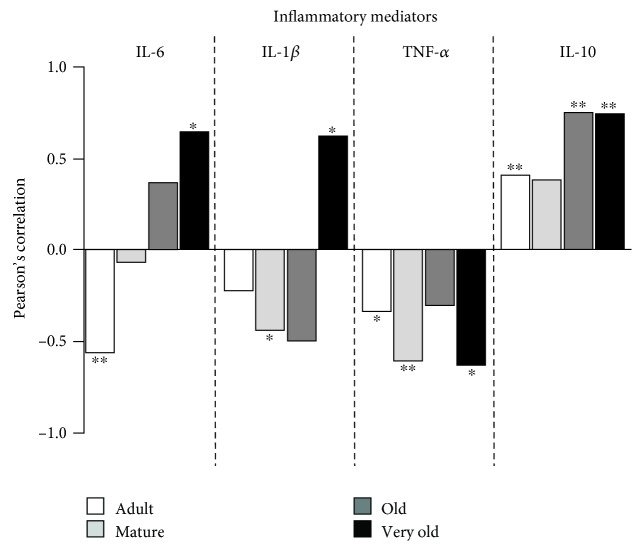
Pearson's correlation coefficient for each cytokine basal release and lifespan at each age. Adult mice: 40 weeks old. Mature mice: 56 weeks old. Old mice: 72 weeks old. Very old mice: 96 weeks old. ^∗^*P* < 0.05; ^∗∗^*P* < 0.01; ^∗∗∗^*P* < 0.001.

With respect to the inflammatory mediators (Figure 6), there is a negative correlation between basal release of IL-6 and lifespan at the adult age (*P* < 0.01) and a positive correlation between basal release of IL-6 and lifespan at the very old age (*P* < 0.05). Likewise, a negative correlation was found between IL-1*β* and lifespan at the mature age (*P* < 0.05) whereas a positive correlation was found between IL-1*β* and lifespan at the very old age (*P* < 0.05). For TNF-*α*, there is a negative correlation between its basal release and achieved lifespan at the adult, mature, and very old ages (*P* < 0.05; *P* < 0.01; and *P* < 0.05, respectively), whereas for IL-10 there is a positive correlation with lifespan at the adult, old, and very old ages (*P* < 0.01).

## 4. Discussion

Biomarkers of aging are defined by their quality of predicting life expectancy, but given that life expectancy data for many individuals are difficult to collect, most of the studies establish “biomarkers of age” instead of biomarkers of aging. The “biomarkers of age” concept is simply based on cross-sectional trends of features as a function of time. According to [13], “the regular and progressive changes over time *per se* do not constitute aging unless they produce some deleterious outcome.” Thus, using longitudinal evidence, biomarkers of age can be validated as biomarkers of aging if high or low values are associated with deleterious or beneficial effects [14, 15]. The present longitudinal study shows that several function, redox, and inflammatory parameters of immune cells are strongly related to lifespan. Nevertheless, the most outstanding contribution is that this relationship with lifespan is different for each parameter depending on the age of the subject. Thus, our results show that certain parameters, such as the ability to proliferate and migrate towards a stimulus of lymphocytes, the capacity to migrate and ingest foreign particles of macrophages, the GPx activity, concentration of GSH and MDA as well as the basal release of IL-6, TNF-*α* and IL-10, strongly correlate with the final achieved lifespan of each individual mouse at the adult age. The early power of these parameters in forecasting lifespan highlights their essential role for the maintenance of health throughout aging, and therefore, they could be used as biomarkers of the rate of aging, i.e., of biological age. In addition, some of these parameters, such as macrophage chemotaxis and phagocytosis, GPx activity and basal release of TNF-*α* and IL-10, are also predictive of lifespan at very old age, and thus, they could be investigated in old animals in order to discriminate which will reach extreme longevity. The fact that macrophage function relates to lifespan across lifetime supports the hypothesis of phagocytes being the main cells responsible for the chronic oxidative and inflammatory stress associated with immunosenescence and therefore responsible for the rate of aging of a subject [6, 16, 17]. In addition, the strong relation to lifespan of GPx activity during aging could be due to its ability to disarm hydrogen peroxide, limiting its harmful effects and therefore playing a critical role against oxidative stress establishment. It could also be related to its ability to inhibit degradation of the inhibitory subunit *α* of nuclear factor-kappa b (NF-kB) [18], and thus, decreasing NF-kB activation, this enzyme could counteract inflammatory stress. In fact, in a previous study, it was demonstrated that those mice which reach high longevity had a low level of basal NF-kB activation [19]. In accordance with that, it was found in the present study that the lower the release of the proinflammatory TNF-*α* and the higher the release of the anti-inflammatory IL-10 during aging, the greater the chance of achieving higher longevity. These results agree with a previous study in which a low basal release of TNF-*α* and a high release of IL-10 were reported in extremely long-lived mice [20] and with another one in which the IL-10/TNF-*α* ratio was proposed as a marker of longevity in mice [21].

Furthermore, another striking finding of our study is that there are some parameters which were not associated with longevity at the adult age, but they become strong discriminant parameters for reaching extreme longevity at the very old age. This is the case for the natural killer (NK) cytotoxic activity and CAT and GR activities as well as the basal release of IL-6 and IL-1*β*. This fact probably indicates that these markers reflect some underlying mechanisms, which are needed to reach high longevity. With respect to NK cytotoxicity and antioxidant CAT and GR activities, there seems to be a “biological purpose.” The higher the NK cytotoxic activity against tumor cells and the higher the decomposition of ROS at very old age, the higher the possibility of becoming long-lived. However, what is the “biological purpose” of an increased IL-6 and IL-1*β* release at very old age if it exacerbates the chronic inflammatory stress? One potential explanation could be that the age-related increase in proinflammatory cytokines is delayed in long-lived mice, this being the reason why they show a high release of both cytokines at very old age. Another possibility could be that these cytokines might be playing an essential role regulating and orchestrating other cells given that only those mice which are able to increase their basal release at the very old age are the ones that later on reach high longevity. Accordingly, recent studies have unravelled a new role of IL-6 in mediating the reprogramming of cells associated with senescence [22], which could reverse the age-associated silencing of important antioxidant and anti-inflammatory genes.

In addition, another intriguing discovery of the study is that certain parameters can be prolongevity or antilongevity depending on the age window studied. In the present study, the NK cytotoxicity and the CAT and GR activities as well as the basal release of IL-6 and IL-1*β* were negatively associated with longevity at the adult age. However, at the very old age, their significance totally changes, the higher these parameters the higher the lifespan achieved. Thus, these parameters seem to exhibit a reverse antagonistic pleiotropy. In fact, there are some other examples of markers whose roles (early antilongevity, late prolongevity) also change during aging such as the systolic blood pressure [23–26] or body mass index [27, 28]. Therefore, these results underscore the necessity to take into account the age of the individual when investigating the predictive power of a given parameter towards longevity. Moreover, they also have important practical applications, as several strategies designed to promote longevity may be useful, useless, or harmful depending on the age at which they are carried out, as previously suggested [14]. In agreement with the results of the present study, overexpression of mitochondrial CAT in young mice was found to be detrimental, whereas its overexpression in old mice had a positive effect [29]. Thus, it seems that ROS exhibit conventional antagonistic pleiotropy explaining why stronger antioxidant mechanisms have not evolved under natural selection of young animals in nature.

Finally, our study is the first one to show the age-associated changes, regarding function, redox and inflammatory parameters in immune cells, which a long-lived individual experiences from adulthood to death. It has been proposed that long-lived individuals have a slower or decelerated aging rate [30], maintaining optimal cell functioning through aging. Strikingly, the results from our study reveal that long-lived subjects do not maintain optimal functioning, redox, and inflammatory state of their leukocytes throughout their aging. In fact, they also experience an age-associated impairment when they are old, although slighter than the one suffered by those who do not reach these advanced ages. However, they are able to adapt and rearrange these parameters at the very old age, showing optimal levels when they are long-lived. It seems that those individuals which show more biological plasticity or adaptive homeostasis [31] by restoring or even increasing cytotoxic NK activity and macrophage functions, antioxidant enzyme activities (CAT, GPx, and GR activities), and basal release of cytokines (IL-6, IL-1*β*, IL-10, and TNF-*α*) at the very old age achieve higher longevity. The most important limitation of the work is the small sample size of mice that reached extreme longevity, and therefore, the results of the present study should be validated using a larger number of mice. In addition, the observed relationships between these parameters and longevity should also be investigated in males, given that female mice, in addition to having a longer lifespan than male mice, also display a different immunity and oxidative and inflammatory states, among other characteristics [32]. Therefore, it seems plausible that those markers that are predictive of longevity in female mice could be different to those in males. Nevertheless, although preliminary, the results of the present study provide a new perspective on the use of function, redox, and inflammatory parameters of immune cells as prognostic tools in aging research and represent a novel benchmark for future work aimed at prediction of lifespan. Moreover, given that the function and oxidative stress of immune cells have been shown to follow a similar pattern in humans and mice [11, 12], strategies that are aimed at boosting the NK cytotoxicity, macrophage functions, and antioxidant enzyme activities as well as at controlling basal cytokine release in elderly individuals could help in achieving healthy aging for the general population.

## Data Availability

The data used to support the findings of this study are available from the corresponding author upon request.
